# An Exercise Mimetic Approach to Reduce Poststroke Deconditioning and Enhance Stroke Recovery

**DOI:** 10.1177/15459683211005019

**Published:** 2021-04-07

**Authors:** Matthew W. McDonald, Matthew S. Jeffers, Lama Issa, Anthony Carter, Allyson Ripley, Lydia M. Kuhl, Cameron Morse, Cesar H. Comin, Bernard J. Jasmin, Baptiste Lacoste, Dale Corbett

**Affiliations:** 1 University of Ottawa, ON, Canada; 2 Canadian Partnership for Stroke Recovery, Ottawa, ON, Canada; 3 Ottawa Hospital Research Institute, ON, Canada; 4 Federal University of São Carlos, SP, Brazil; 5 University of Ottawa Brain and Mind Research Institute, ON, Canada

**Keywords:** aerobic exercise, resistance exercise, skeletal muscle, angiogenesis, cardiovascular

## Abstract

Evidence supports early rehabilitation after stroke to limit disability. However, stroke survivors are typically sedentary and experience significant cardiovascular and muscular deconditioning. Despite growing consensus that preclinical and clinical stroke recovery research should be aligned, there have been few attempts to incorporate cardiovascular and skeletal muscle deconditioning into animal models of stroke. Here, we demonstrate in rats that a hindlimb sensorimotor cortex stroke results in both cardiovascular and skeletal muscle deconditioning and impairments in gait akin to those observed in humans. To reduce poststroke behavioral, cardiovascular, and skeletal muscle perturbations, we then used a combinatorial intervention consisting of aerobic and resistance exercise in conjunction with administration of resveratrol (RESV), a drug with exercise mimetic properties. A combination of aerobic and resistance exercise mitigated decreases in cardiovascular fitness and attenuated skeletal muscle abnormalities. RESV, beginning 24 hours poststroke, reduced acute hindlimb impairments, improved recovery in hindlimb function, increased vascular density in the perilesional cortex, and attenuated skeletal muscle fiber changes. Early RESV treatment and aerobic and resistance exercise independently provided poststroke benefits, at a time when individuals are rapidly becoming deconditioned as a result of inactivity. Although no additive effects were observed in these experiments, this approach represents a promising strategy to reduce poststroke behavioral impairments and minimize deconditioning. As such, this treatment regime has potential for enabling patients to engage in more intensive rehabilitation at an earlier time following stroke when mechanisms of neuroplasticity are most prevalent.

## Introduction

With advances in acute care, stroke has transitioned from a disease of survivability to one of chronic disability.^1^ To optimize behavioral recovery, preclinical evidence suggests that high-intensity rehabilitation should be initiated during the early subacute stroke recovery phase to harness the brain’s endogenous repair mechanisms.^2-5^ Instead, at this time stroke patients are extremely inactive, spending more than 50% of their time lying in bed and ~87% of time in a sedentary state.^6,7^ As such, stroke survivors experience significant deconditioning and fatigue, which reduces their capacity to engage in high-intensity rehabilitation and accomplish most activities of daily living.^8-11^

International consensus groups recognize that preclinical and clinical research must become better aligned to advance the field of stroke recovery.^12,13^ In humans, the stroke-affected limb undergoes significant muscular atrophy, hypertrophy of slow-twitch fibers, and atrophy of fast-twitch fibers.^8,9,14^ Similarly, stroke survivors experience significant cardiovascular deconditioning.^8-11^ Currently, these aspects of cardiovascular and skeletal muscle dysfunction have not been incorporated into animal models of stroke, nor has consideration been given to the idea that the benefits of poststroke interventions might be a result of central as well as peripheral actions.^8,9,11,14,15^

Exercise is viewed as a powerful poststroke therapeutic, enhancing brain repair through multiple mechanisms such angiogenesis, upregulation of growth factors, reducing inflammation, and attenuating deteriorations in cardiovascular and skeletal muscle health.^11,16,17^ However, implementing aerobic exercise following stroke is challenging, largely because of fatigue and deconditioning.^8,18^ Thus, poststroke deconditioning is a significant barrier that limits patients’ ability to engage in early, intensive rehabilitation programs. A possible solution is to administer drugs that induce central and peripheral effects similar to physical exercise (ie, exercise mimetics). In this context, resveratrol (RESV), a naturally occurring polyphenol (found in grapes, blueberries, peanuts, etc), has the potential to enhance neurological recovery and attenuate poststroke cardiovascular and skeletal muscle deconditioning by activation of sirtuin 1 (SIRT1), peroxisome proliferator-activated receptor gamma coactivator 1-α (PGC1α), and angiogenesis in a variety of body tissues (eg, brain, heart, muscle) similar to exercise.^19-23^

Because lack of mobility is such an important issue for patients following stroke, we used a rat model of hindlimb motor cortex stroke to assess poststroke changes in skeletal muscle and cardiovascular deconditioning. We hypothesized that both RESV and delayed aerobic and resistance exercise (DEx) following stroke would enhance behavioral recovery and attenuate poststroke deconditioning but that the combination would be most effective. In summary, this study aimed to address a number of interrelated research questions: (1) In conjunction with persistent hindlimb deficits, do rats experience cardiovascular deconditioning and skeletal muscle fiber changes as observed clinically? (2) Does early RESV administration reduce acute hindlimb deficits? (3) Does RESV alone or in combination with exercise rehabilitation improve long-term hindlimb recovery? (4) Does RESV alone or in combination with exercise mitigate perturbations in cardiovascular fitness and skeletal muscle after stroke?

## Methods

### Animals

**Figure 1.**
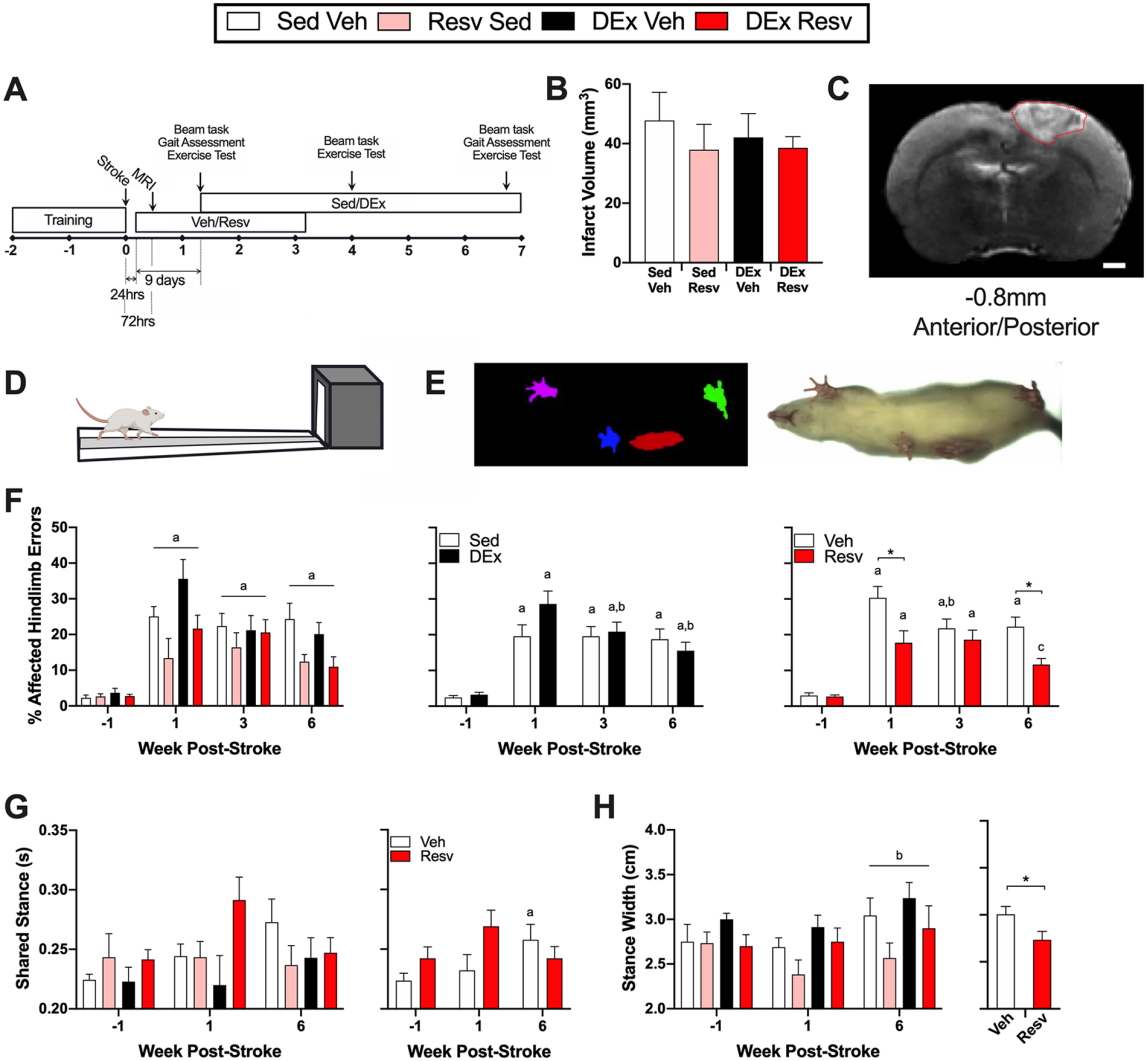
(A) Experimental timeline. (B) Infarct volume. (C) Representative magnetic resonance image of median infarct; stroke delineated in red; 1000-µm scale bar. Hindlimb function was assessed on the (D) beam traversal task and (E) through kinematic assessment of gait. (F) Left panel: stroke resulted in increased foot faults on the beam traversal task over time (n = 9). Middle panel: exercise rehabilitation improved beam performance (Time × DEx; n = 16-18). Right panel: resveratrol resulted in less foot faults at week 1 and week 6 poststroke (Time × Resv; n = 16-18). (G) Left panel: duration of time spent in shared stance during gait (n = 6-9). Right panel: Resv prevented the development of increased shared stance time (Time × Resv; n = 15-17). (H) Left panel: rats developed a wider stance width at week 6 compared with week 1 poststroke (n = 6-9). Right panel: overall, rats that received Resv had a narrower stance width (n = 29-32). Abbreviations: Resv, resveratrol; Sed Veh, sedentary vehicle control; Sed Resv, sedentary Resv; DEx Veh, exercise vehicle; DEx Resv, exercise Resv. ^a^ Different from prestroke. ^b^ Different from 1 week poststroke. ^c^ Different from 3 weeks poststroke. * *P* < .05.

Female Sprague-Dawley rats (n = 40; 200-210 g) from Charles River Laboratories (Montreal, QC, Canada) were pair housed in standard cages on a 12-hour light/dark cycle at a constant temperature and relative humidity. Rats had access to water and standard chow ad libitum. All animal procedures were approved by the Animal Care Committee of the University of Ottawa in accordance with the guidelines of the Canadian Council on Animal Care. Rats were randomly allocated to 1 of 4 stroke groups (n = 10/group): sedentary vehicle control (Sed Veh; n = 9), sedentary RESV (Sed RESV; n = 8), exercise vehicle (DEx Veh; n = 9), or exercise RESV (DEx RESV; n = 9). Five rats were excluded from the experiment (n = 2 died during surgery, and n = 3 had a failed stroke surgery). Figure 1A shows the experimental timeline.

### Stroke Induction

Strokes were induced via photothrombosis after a 12-hour fast. Briefly, rats were anesthetized (4% isoflurane induction, 2% isoflurane maintenance) and injected with rose Bengal through a lateral tail vein catheter (Sigma, R3877; 20 mg/kg; dissolved in sterile water); after 2 minutes, a cold light was illuminated for 10 minutes over the hindlimb sensorimotor cortex (anterior-posterior = −1.5 mm, medial-lateral = ±3 mm, relative to Bregma).

### Resveratrol

At 24 hours after stroke when cell death is complete,^24^ rats received a 5-mg/kg injection (intraperitoneal) of RESV (Toronto Research Chemicals Inc, R150000) or vehicle (20% β-cyclodextrin, Toronto Research Chemicals Inc, H952565; 0.9% NaCl). RESV treatment continued daily for 3 weeks. RESV dose was based on past efficacy^22^ and a pilot dose-response experiment (see Supplemental Materials, Figure 1).

### Magnetic Resonance Imaging

Infarct volume was determined 72 hours after stroke with a small animal magnetic resonance scanner (Agilent MR901 7T, General Electric, USA). Rats were anesthetized with isoflurane as above and vital signs and body temperature were monitored (SA Instruments Inc, Stony Brook, USA). Images were acquired with a T2-weighted fast spin echo pulse sequence: 15 axial (transverse) slices; slice thickness = 800 µm; in-plane resolution = 78 µm; echo train length = 8; echo time = 27 ms; scan time = 5 minutes. Infarcts were outlined using ImageJ (National Institute of Health Research, USA) by an experimenter blinded to experimental groups and the volumes determined by multiplying the infarcted area for all coronal sections by magnetic resonance slice thickness.

### Exercise Rehabilitation

**Figure 2.**
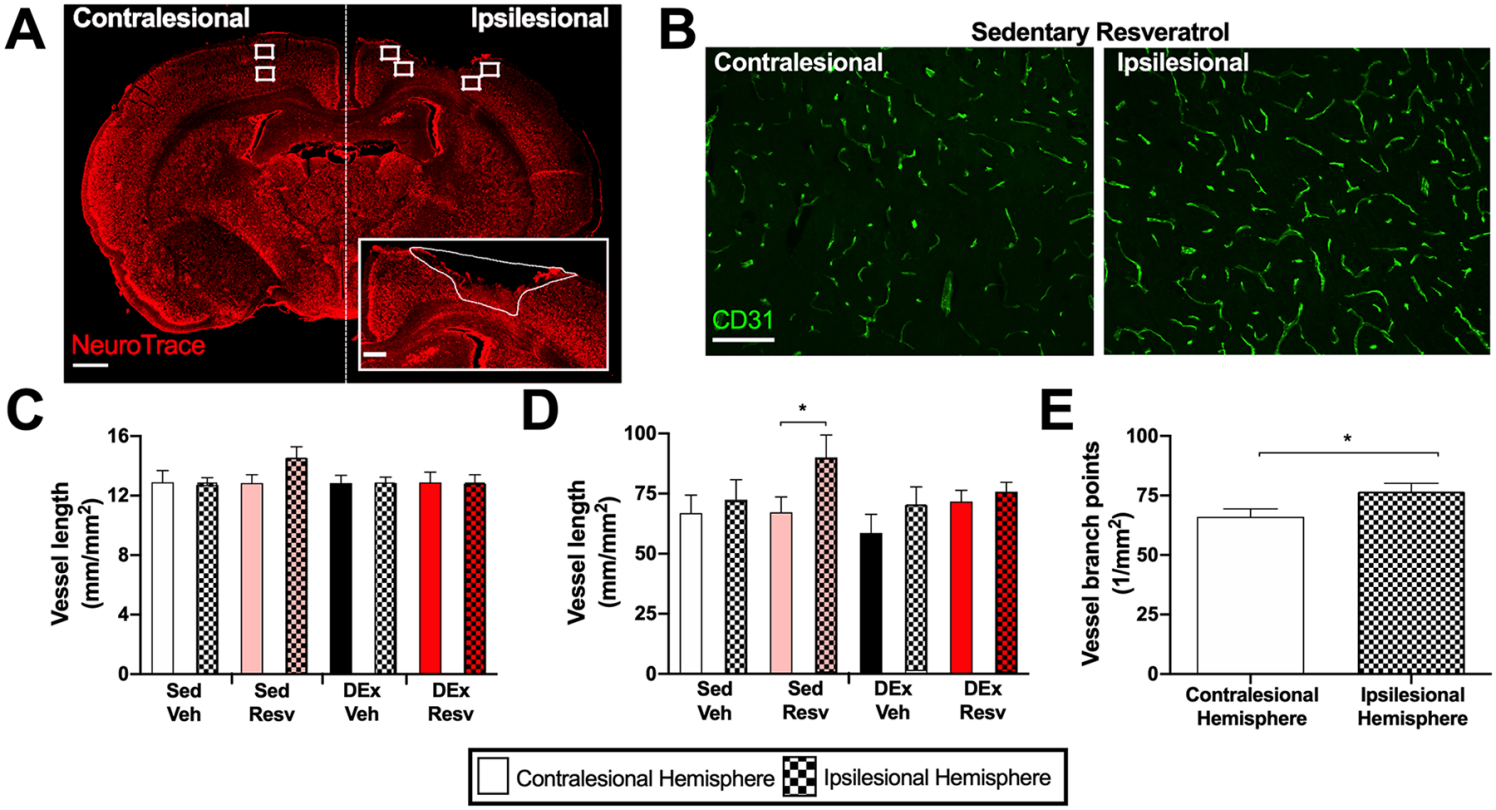
Resveratrol increased vascular density in the ipsilesional hemisphere. (A) Representative image of infarct with NeuroTrace (red) and ROI areas that were sampled; 1000-µm scale bar. Inset: Representative image of delineated infarct area; 250-µm scale bar. (B) Representative CD31 (green) images within the sedentary Resv group; 100-µm scale bar. (C) Vessel length in the cortex was similar between groups and independent of hemisphere (n = 7-9). (D) Sedentary Resv had more vascular branch points in the ipsilesional compared with the contralesional hemisphere (n = 7-9). (E) Vascular branch points were increased in the ipsilesional hemisphere compared with the contralesional hemisphere (n = 34). Abbreviations: Resv, resveratrol; Sed Veh, sedentary vehicle control; Sed Resv, sedentary Resv; DEx Veh, exercise vehicle; DEx Resv, exercise Resv; ROI, region of interest. * P < .05.

Beginning 10 days poststroke, exercise rehabilitation (Figures 1A and 2C) consisting of either aerobic or resistance training on alternating days (5 d/wk) was delivered for 5 weeks. Aerobic exercise was conducted on a motorized treadmill for 45 minutes at 70% to 80% of each rat’s peak oxygen consumption (VO_2_ peak; measured 10 days and 4 weeks poststroke), in accordance with physical activity and exercise recommendations for stroke survivors.^25^ For resistance exercise (see Supplemental Material for detailed protocol^26,27^), rats climbed a vertical ladder with 50%, 75%, 90%, and 100% of their maximal lifting capacity for the first 4 climbs, followed by 100% of their maximal lifting capacity until exhaustion (~8-12 climbs).

### Beam Traversal Task

Rats were trained to walk across a tapered beam (length 90 cm; width 6-1.5 cm) to a dark goal box containing 3 food pellets (TestDiet, 45 mg; Figure 1D). The beam traversal task was conducted prestroke and at 1 week, 4 weeks, and 6 weeks poststroke. Rats were filmed for 4 trials, and the percentage of foot faults was determined by dividing the number of foot faults by the total number of steps.

### Gait Assessment

Gait was assessed by ventral plane videography using the DigiGait System (Mouse Specifics Inc, USA) to generate digital paw placements (Figure 1E). Prior to testing, rats were familiarized to walking on a treadmill over 1 week. Videos (150 frames/s) were recorded from below as rats walked on a transparent motor-driven treadmill at 26 cm/s. Gait indices were determined using DigiGait software that plotted the area of each paw in contact with the treadmill belt over time. Gait was assessed prestroke and at 1 week and 6 weeks poststroke.

### Maximal Exercise Test

VO_2_ peak during a maximal exercise test (see Supplemental Methods) was measured prestroke and at 1 week, 4 weeks, and 6 weeks poststroke on a metabolic treadmill equipped with an OxyMax system (Columbus Instruments, USA). Because of difficulties with running compliance during repetitive maximal exercise testing, VO_2_ peak measures at week 6 were not obtained in all animals (n = 1 Sed Veh; n = 4 Sed RESV; n = 1 DEx Veh). These animals were excluded from maximal exercise test analysis but included for all other measures. Distance to exhaustion was also determined for each test session.

### Tissue Preparation

At 24 hours after their last exercise bout, rats were anesthetized with 65 mg/mL euthanyl (intraperitoneal). Rats were perfused with heparinized saline, and the tibialis anterior and plantaris were removed. The plantaris muscles were embedded in optimal cutting temperature medium (Tissue-Tek, Sakura Finetek USA Inc), frozen in liquid nitrogen cooled isopentane, and stored at −80 °C prior to cryosectioning at 20 µm. The tibialis anterior was frozen in liquid nitrogen and stored at −80 °C. Subsequently, the descending aorta was clamped and rats were perfused with cold 4% paraformaldehyde (PFA) in phosphate-buffered saline (PBS). Brains were postfixed in PFA overnight at 4 °C, placed in 30% sucrose at 4 °C until saturated, and cryosectioned at 20 µm (1:24 intervals).

### Brain Immunofluorescence

Brain sections were incubated with a blocking solution (BS; 10% goat serum, PBS) for 1 hour at room temperature followed by overnight incubation of BS with CD31 primary antibody (AF3628, 1:200) from R&D Systems (USA). Sections were then washed in PBS, incubated for 1 hour in secondary antibody (Alexa Fluor 488, 1:300) and NeuroTrace (640/680; 1:150) from ThermoFisher Scientific (USA), washed in PBS, and cover slipped with aqueous mounting medium (Immu-Mount, Thermo Scientific). Regions of interest adjacent to both the lesioned and contralesional cortex were imaged using an Axio Observer Z1 with the Apotome 2 module (Carl Zeiss). Blood vessel density was calculated as the sum of the lengths of all blood vessel segments in the image divided by the imaged area. The branching point density was computed as the number of branching points divided by the image area (see Supplemental Methods).

### Skeletal Muscle Immunofluorescence and Western Blotting

Muscle fiber staining procedures were adapted from previous publications.^28^ Muscle sections were incubated with a BS (10% goat serum, PBS) for 1 hour at room temperature followed by overnight incubation of BS with primary antibodies for myosin heavy chain (MHC) I (BA-F8, 1:50), MHCIIa (SC-71, 1:600), and MHCIIb (BF-F3, 1:100) from the Developmental Studies Hybridoma Bank at the University of Iowa, as well as laminin (ab1157, 1:450) from Abcam (USA) at 4 °C. The next day, sections were washed in PBS, incubated at room temperature for 1 hour in BS and secondary antibodies (Alexa Fluor 350 IgG2b, 1:500; Alexa Fluor 488 IgG1, 1:500; Alexa Fluor 555 IgM, 1:500; and Alexa Fluor 647 IgG, 1:300) from Invitrogen (USA), washed in PBS, and coverslipped with aqueous mounting medium (Immu-Mount, Thermo Scientific). To assess vascular density, skeletal muscle sections were incubated in BS for 1 hour, followed by 90-minute incubation at room temperature in BS with CD31 primary antibody (AF3628, 1:200) from R&D Systems (USA) and laminin (ab1157, 1:450) from Abcam (USA) to identify muscle fibers. Sections were then washed in PBS, followed by 1-hour incubation with secondary antibodies (Alexa Fluor 488, 1:300; Alexa Fluor 680, 1:300) from ThermoFisher Scientific, washed in PBS, and coverslipped with aqueous mounting medium (Immu-Mount, Thermo Scientific). Muscle sections were visualized with an Axio Observer Z1 microscope (Carl Zeiss) using conventional fluorescence microscopy under 10× (muscle fiber type quantification) and 20× (vascular density) magnification. Muscle fibers and CD31-labeled capillaries were quantified using the neural network software Ilastik (www.ilastik.org) and a custom ImageJ script (see Supplemental Methods). To assess the aerobic capacity of skeletal muscle, the amount of PGC1α protein in the tibialis anterior muscle from the affected limb was assessed through Western blotting (see Supplemental Methods).

### Statistical Analysis

All statistical analyses were performed using SPSS (IBM Corp, Armonk, NY). Infarct volume and distance to exhaustion during maximal exercise test were compared using a 2-way (between-subject variables: RESV × DEx) analysis of variance (ANOVA). Beam traversal, DigiGait, and metabolic treadmill data were compared using a 3-way ANOVA (within-subjects variable: time; between-subject variables: RESV × DEx). Muscle mass, fiber cross-sectional area (CSA), fiber type proportions, and brain CD31 were compared using a 3-way ANOVA (within-subjects variables: affected limb or hemisphere; between-subjects variables: RESV × DEx). When assumptions of sphericity were violated, the Greenhouse-Geisser correction was applied. Sidak-corrected *t*-tests were used to correct for multiple comparisons. The relationship between vascular density measures and beam traversal performance was investigated by determining the Pearson correlation coefficient. Frequency distributions of muscle fiber CSA were compared using a Kruskal-Wallis test. When a significant difference was detected, a stepwise test was used to identify homogeneous subsets. For all statistical comparisons, *P* <.05 was deemed significant. All data except those presented in Table 1 are presented as the mean ± standard error. Table 1 represents the mean ± SD of each gait index.

**Table 1.** Gait Indices in the Affected Hindlimb Over Time.

		Prestroke				1 Week poststroke				6 Weeks poststroke			
		Sed Veh	Sed RESV	DEx Veh	DEx RESV	Sed Veh	Sed RESV	DEx Veh	DEx RESV	Sed Veh	Sed RESV	DEx Veh	DEx RESV
Affected Hindlimb	Brake (s)	0.04 ± 0.02	0.05 ± 0.02	0.04 ± 0.02	0.05 ± 0.02	0.06 ± 0.04^a^	0.07 ± 0.02^a^	0.07 ± 0.02^a^	0.07 ± 0.03^a^	0.05 ± 0.02^a^	0.06 ± 0.02^a^	0.07 ± 0.03^a^	0.07 ± 0.03^a^
	Maximum d*A*/d*T* (cm^2^/s)	517.04 ± 83.4	605.61 ± 61.88	577.61 ± 116.41	544.17 ± 74.89	436.14 ± 120.50^a^	471.24 ± 40.59^a^	426.93 ± 116.43^a^	453.43 ± 80.49^a^	469.79 ± 99.06^a^	440.57 ± 88.93^a^	468.89 ± 102.84^a^	451.26 ± 38.65^a^
	Minimum d*A*/d*T* (cm^2^/s)	−57.31 ± 21.44	−65.33 ± 14.95	−65.84 ± 21.44	−59.62 ± 13.95	−55.18 ± 10.95	−61.16 ± 12.88	−60.47 ± 29.18	−52.43 ± 16.29	−55.25 ± 13.76	−57.17 ± 17.88	−69.91 ± 24.99	−75.05 ± 26.55
	Overlap distance (cm)	−0.37 ± 3.12	−2.09 ± 7.43	−0.84 ± 4.96	0.47 ± 1.21	−3.52 ± 7.49	−1.8 ± 2.56	−9.6 ± 13.36	−4.7 ± 7.94	0.14 ± 1.70	−3.56 ± 4.20	−2.76 ± 6.67	−0.62 ± 2.69
	Paw angle variability (degrees)	6.6 ± 1.15	6.62 ± 2.26	5.5 ± 2.20	6.2 ± 2.85	5.68 ± 1.92	6.52 ± 2.09	7.27 ± 2.66	6.07 ± 3.04	5.18 ± 2.26	6.13 ± 1.97	7.89 ± 1.95	9.04 ± 3.17
	Paw area at peak stance (cm^2^)	4.93 ± 0.77	5.92 ± 0.72	5.5 ± 1.07	5.31 ± 0.77	4.44 ± 1.00^a^	4.81 ± 0.41^a^	4.43 ± 1.00^a^	4.59 ± 0.69^a^	4.94 ± 1.06	4.7 ± 1.12	5.02 ± 0.91	4.92 ± 0.41
	Propel (s)	0.3 ± 0.03	0.31 ± 0.03	0.29 ± 0.03	0.31 ± 0.04	0.31 ± 0.04	0.3 ± 0.04	0.29 ± 0.03	0.33 ± 0.06	0.34 ± 0.04	0.31 ± 0.02	0.3 ± 0.03	0.3 ± 0.04
	Stance (s)	0.34 ± 0.02	0.36 ± 0.05	0.33 ± 0.03	0.36 ± 0.03	0.37 ± 0.03^a^	0.37 ± 0.05^a^	0.36 ± 0.04^a^	0.4 ± 0.05^a^	0.39 ± 0.05^a^	0.37 ± 0.03^a^	0.37 ± 0.04^a^	0.37 ± 0.03^a^
	Stride (s)	0.47 ± 0.02	0.48 ± 0.06	0.46 ± 0.05	0.49 ± 0.04	0.53 ± 0.05^a^	0.51 ± 0.07^a^	0.51 ± 0.05^a^	0.54 ± 0.06^a^	0.54 ± 0.06^a^	0.54 ± 0.03^a^	0.53 ± 0.06^a^	0.54 ± 0.03^a^
	Stride length (cm)	12.1 ± 0.54	12.62 ± 1.58	11.99 ± 1.18	12.77 ± 1.00	13.85 ± 1.31^a^	13.22 ± 1.79^a^	13.24 ± 1.25^a^	13.97 ± 1.45^a^	14.11 ± 1.53^a^	14 ± 0.78^a^	13.67 ± 1.43^a^	13.91 ± 0.85^a^
	Swing (s)	0.12 ± 0.01	0.12 ± 0.01	0.13 ± 0.02	0.13 ± 0.01	0.17 ± 0.04^a^	0.14 ± 0.03^a^	0.15 ± 0.06^a^	0.14 ± 0.01^a^	0.15 ± 0.05^a^	0.17 ± 0.03^a^	0.16 ± 0.03^a^	0.17 ± 0.03^a^
Both Hindlimbs	Stance width (cm)	2.81 ± 0.56	2.73 ± 0.31^b^	2.97 ± 0.20	2.57 ± 0.26^b^	2.73 ± 0.30	2.38 ± 0.40^b^	2.81 ± 0.28	2.73 ± 0.46^b^	3.04 ± 0.52^c^	2.57 ± 0.41^b,c^	3.33 ± 0.45^c^	2.83 ± 0.74^b,c^
	Stance width variability (cm)	0.52 ± 0.12	0.48 ± 0.21	0.41 ± 0.10	0.47 ± 0.04	0.65 ± 0.24^a^	0.53 ± 0.15^a^	0.55 ± 0.17^a^	0.55 ± 0.08^a^	0.49 ± 0.19	0.52 ± 0.18	0.41 ± 0.14	0.49 ± 0.08
	Shared stance (s)	0.22 ± 0.01	0.24 ± 0.05	0.22 ± 0.03	0.24 ± 0.02	0.24 ± 0.03	0.25 ± 0.03	0.22 ± 0.06	0.29 ± 0.05	0.27 ± 0.05	0.24 ± 0.04^a^	0.24 ± 0.04	0.25 ± 0.03^a^

Abbreviations: RESV, resveratrol; Sed Veh, sedentary vehicle control; Sed RESV, sedentary RESV; DEx Veh, exercise vehicle; DEx RESV, exercise RESV.

a different from prestroke.

b main effect of Resv, independent of time and DEx.

c different from 1 week poststoke.

## Results

### Similar Lesion Volumes in All Experimental Groups

Hindlimb sensorimotor cortex stroke induced by cold light photothrombosis (Figure 1C) resulted in similar lesion volumes (Figure 1B) in all groups, independent of RESV (*F*_1,31_ = 0.745; *P* = .395) and exercise (*F*_1,31_ = 0.104; *P* = .749), indicating that outcome differences occurred independent of lesion size.

### Stroke Produces Consistent Hindlimb Gait Deficits

Stroke increased beam foot faults independent of experimental group (*F*_3,93_ = 8.364, *P* < .001; Figure 1F, left panel). Compared with prestroke, foot faults were increased at week 1 (*t*_35_ = 48.831; *P* < .0001), week 3 (*t*_35_ = 9.251; *P* < .0001), and week 6 (*t*_35_ = 7.645; *P* < .0001) following stroke. Gait deficits in the affected hindlimb were associated with abnormalities in coordination (Table 1). Following stroke, paw area at peak stance (the capacity to load the limb) changed over time (*F*_2,48_ = 7.489; *P* = .001), where area decreased at week 1 poststroke (*t*_27_ = 3.965; *P* = .002) and normalized by week 6 (*t*_27_ = 1.924; *P* = .182). Similarly, variability in stance width changed over time (*F*_2,46_ = 4.762; *P* = .013), increasing at week 1 poststroke (*t*_27_ = 3.300; *P* = .008) and normalizing by week 6 (*t*_27_ = 0.136; *P* = .999). Stroke also caused other persistent deficits in the affected limb, indicating that gait was slow, inconsistent, and exaggerated. Specifically, at week 1 and week 6 poststroke, the duration of time in stance, swing phase, brake phase, stride phase, and stride length were increased, and speed of loading the affected limb was decreased (see Supplemental Materials for statistics).

### RESV Reduces Acute Poststroke Hindlimb Deficits

There was an interaction between time poststroke and RESV treatment on the beam traversal task (*F*_3, 93_ = 5.029, *P* = .003; Figure 1F, right panel). RESV treatment beginning 24 hours after stroke reduced acute hindlimb deficits on the beam traversal task at week 1 poststroke (*t*_32.88_ = 2.716, *P* = .041; Figure 1F, right panel).

### RESV Improves Recovery of Hindlimb Function and Increases Vascular Density in the Perilesional Cortex

Rats that received vehicle showed spontaneous recovery on the beam traversal task from week 1 to week 3 poststroke (*t*_17_ = 3.297, *P* = .025; Figure 1F, right panel). Exercise did not improve hindlimb recovery relative to sedentary animals at 6 weeks. However, there was an interaction between time poststroke and exercise on the beam traversal task (*F*_3, 93_ = 3.832; *P* = .012), where the exercise rehabilitation improved beam traversal performance at both week 3 (*t*_17_ = 3.215, *P* = .030; Figure 1F, middle panel) and week 6 (*t*_17_ = 4.939, *P* < .001; Figure 1F, middle panel) compared with 1 week poststroke, when exercise had not yet been initiated. In contrast, RESV improved recovery on the beam task from week 3 to week 6 poststroke (*t*_17_ = 3.265, *P* = .028; Figure 1F, right panel). Importantly, at week 6 poststroke, rats that received RESV made significantly fewer foot faults compared with those that received vehicle (*t*_17_ = 3.293, *P* = .011; Figure 1F, right panel). RESV also prevented the development of compensatory gait strategies. Vehicle-treated rats spent more time in dual support at week 6 compared with week 1 poststroke (*t*_13_ = 2.850, *P* = .040; Figure 1G, right panel), whereas RESV prevented this increase in shared stance time (*t*_12_ = 0.000, *P* > .999; Figure 1G, right panel). Similarly, there was a main effect of RESV on stance width (ie, base of support) during gait (*F*_1,23_ = 5.887, *P* = .024; Figure 1H, right panel), where rats that received only vehicle had a wider base of support.

RESV (*F*_1,29_ = 0.609; *P* = .442), exercise (*F*_1,29_ = 0.505; *P* = .483), and hemisphere (*F*_1,29_ = 1.550; *P* = .223) did not affect vessel length (Figure 2C). In contrast, vessel branch points were increased in the ipsilesional hemisphere compared with the contralesional hemisphere (*F*_1,30_ = 14.02, *P* < .001; Figure 2E). Additionally, there was an interaction between RESV, exercise, and hemisphere (*F*_1,30_ = 4.401, *P* = .044; Figure 2D), where RESV-treated sedentary rats had increased vessel branch points in the ipsilesional hemisphere (*t*_30_ = 3.540, *P* = .037; Figure 2D). Vascular branch points correlated with beam performance at week 1 (*r* = −0.4, *r*^2^ = 0.189; *P* = .011) and week 6 (*r* = −0.3, *r*^2^ = 0.115; *P* = .050).

### Stroke Reduces Cardiovascular Fitness and Exercise Capacity

**Figure 3.**
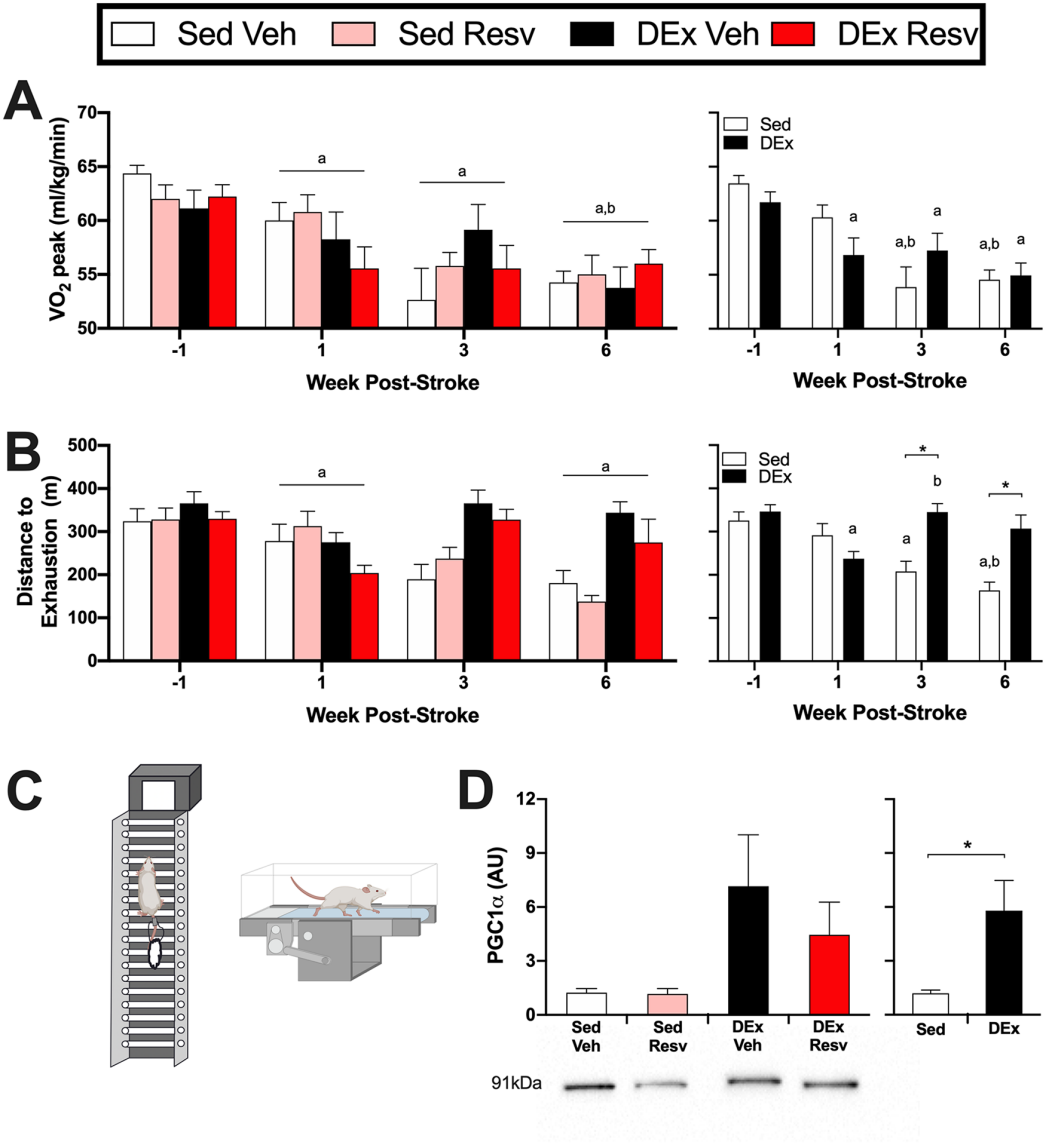
Stroke resulted in decreases in cardiovascular fitness, which was attenuated with delayed exercise. (A) Left panel: stroke reduced VO2 peak during a maximal exercise test (n = 5-9). Right panel: The exercise rehabilitation attenuated further reductions in VO2 peak (Time × DEx; n = 13-17). (B) Left panel: the distance rats could run until exhaustion was reduced at week 1 and week 6 poststroke (n = 5-9). Right panel: rats that engaged in the exercise rehabilitation could run farther until exhaustion at week 3 and week 6 poststroke compared with being sedentary (Time × DEx; n = 13-17). (C) The delayed exercise rehabilitation was a combination of resistance (ladder climbing) and aerobic (treadmill) exercise. (D) Left panel: PGC1α protein content in the red portion of the tibialis anterior (n = 7-8). Right panel: exercise increased PGC1α protein content, indicative of increased aerobic metabolism (n = 15-16). Lower panel: representative Western blot image. Abbreviations: Resv, resveratrol; Sed Veh, sedentary vehicle control; Sed Resv, sedentary Resv; DEx Veh, exercise vehicle; DEx Resv, exercise Resv; VO2 peak, peak oxygen consumption; PGC1α, proliferator-activated receptor gamma coactivator 1-α. ^a^ Different from prestroke. ^b^Different from 1 week poststroke. ^*^P < .05.

Stroke changed VO_2_ peak over time (*F*_3,78_ = 20.296, *P* < .001; Figure 3A, left panel). Compared with prestroke, VO_2_ peak was reduced at week 1 (*t*_29_ = 4.102; *P* = .002), week 3 (*t*_29_ = 5.073; *P* = .001), and week 6 (*t*_29_ = 10.340; *P* < .001) poststroke. Similarly, VO_2_ peak was further reduced at week 6 compared with week 1 poststroke (*t*_29_ = 3.106; *P* = .025). Maximal distance traveled until exhaustion was also reduced after stroke (*F*_2.376,64.140_ = 8.195, *P* < .001; Figure 3B, left panel), where distance to exhaustion was reduced at week 1 (*t*_27_ = 4.669; *P* < .001) and week 6 (*t*_27_ = 3.522; *P* = .009) poststroke.

### Exercise Rehabilitation Mitigates Reductions in Cardiovascular Fitness and Exercise Capacity

There was an interaction between time poststroke and exercise on VO_2_ peak (*F*_3,78_ = 3.838, *P* = .013; Figure 3A, right panel). Peak VO_2_ was reduced in rats that remained sedentary following stroke at week 3 compared with prestroke (*t*_12_ = 4.299; *P* = .006) and week 1 (*t*_12_ = 4.592; *P* = .004) and at week 6 compared with prestroke (*t*_12_ = 6.580; *P* < .001) and week 1 (*t*_12_ = 3.953; *P* = .012). In contrast, rats that engaged in the exercise rehabilitation had a reduction in VO_2_ peak at week 1 (*t*_16_ = 3.305; *P* = .027), week 3 (*t*_16_ = 3.168; *P* = .035), and week 6 (*t*_16_ = 8.791; *P* < .001) poststroke; however, no further reductions in VO_2_ peak were observed at week 3 (*t*_16_ = 0.304; *P* = .999) or week 6 (*t*_16_ = 1.170; *P* = .835) compared with week 1 poststroke. An interaction between time poststroke and exercise on distance traveled to exhaustion was also evident (*F*_2.376,64.140_ = 11.779, *P* < .001; Figure 3B, right panel). Distance sedentary rats could run until exhaustion was reduced at week 3 (*t*_12_ = 5.706; *P* = .008) and week 6 (*t*_12_ = 6.685; *P* = .002) compared with prestroke and at week 6 compared with week 1 (*t*_12_ = 4.547; *P* = .033) poststroke. Rats that engaged in exercise had a reduction in distance traveled until exhaustion at week 1 compared with prestroke (*t*_14_ = 8.498; *P* < .001); however, distance traveled until exhaustion returned to prestroke levels by week 3 (*t*_14_ = 5.580; *P* = .007) poststroke. Importantly, compared with remaining sedentary, rats that engaged in exercise rehabilitation traveled a greater distance until exhaustion at week 3 (*t*_24.18_ = 4.479; *P* < .001) and week 6 (*t*_22.63_ = 3.878; *P* = .003). Indicative of increased aerobic capacity, rats that exercised had increased PGC1α protein content in the red portion of the tibialis anterior muscle compared with those remaining sedentary (*F*_1,26_ = 6.342, *P* = .018; Figure 3D).

### Changes to Skeletal Muscle Fibers Similar to Human Stroke

**Figure 4.**
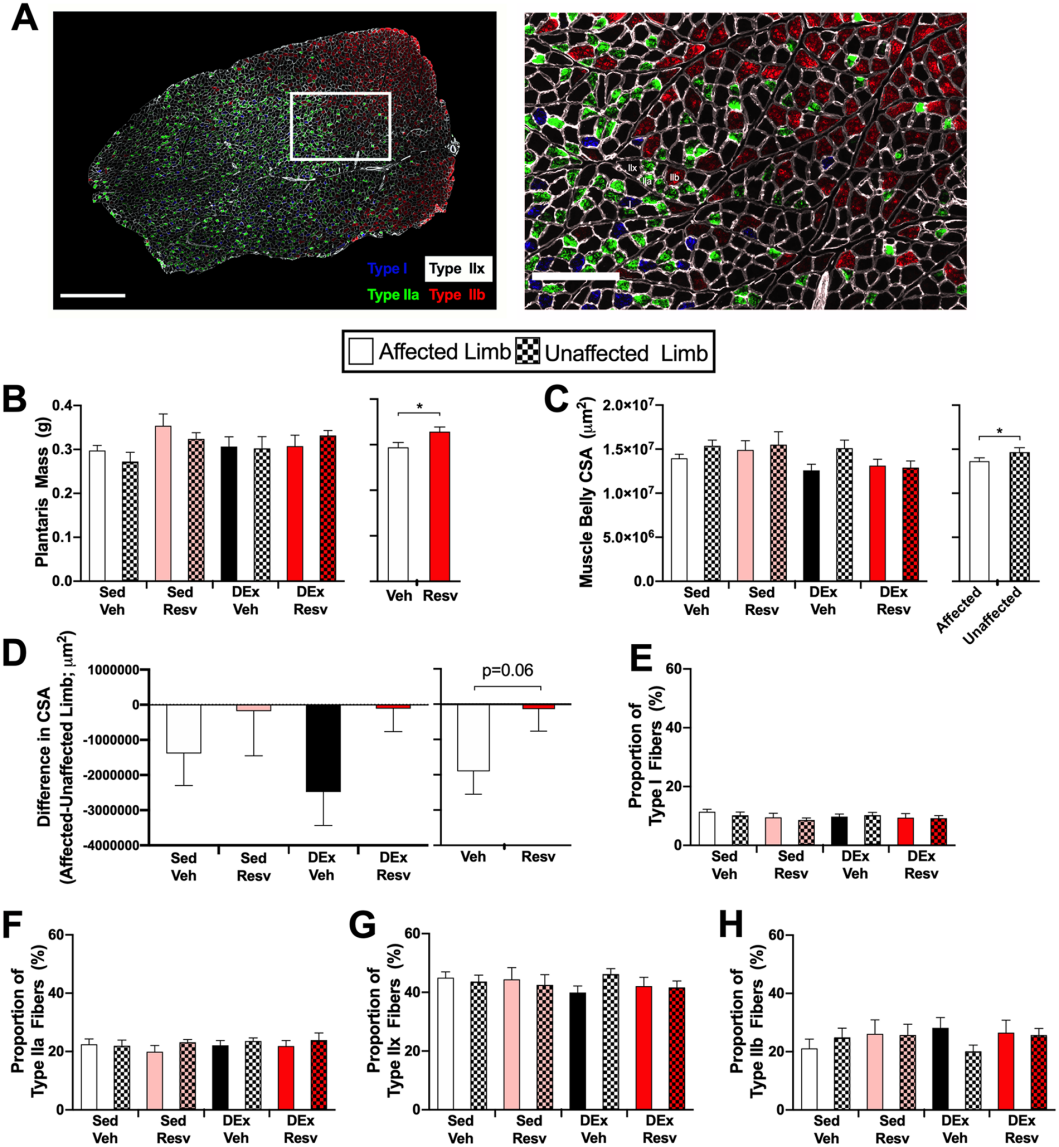
Stroke resulted in mild atrophy throughout the plantaris muscle. (A) Representative image of skeletal muscle fiber types. Left panel: cross section of plantaris muscle with type I (blue), type IIa (green), type IIx (unlabeled), and type IIb (red) muscle fibers; scale bar 1000 µm. Right panel: region depicted in white box; scale bar 300 µm. (B) Left panel: wet mass of plantaris muscle (n = 8-9). Right panel: resveratrol increased the mass of the plantaris muscle independent of limb or exercise. (n = 34-36). (C) Left panel: cross-sectional area (CSA) of plantaris muscle belly (n = 8-9). Right panel: stroke resulted in atrophy of the plantaris muscle in the affected limb independent of Resv or exercise. (D) Left panel: difference in CSA of plantaris muscle between affected and unaffected limb (n = 7-9). Right panel: there was a trend that Resv mitigated atrophy of the affected plantaris muscle (n = 17). (E-H) Proportion of type I, type IIa, type IIx, and type IIb skeletal muscle fibers (n = 8-9). Abbreviations: Resv, resveratrol; Sed Veh, sedentary vehicle control; Sed Resv, sedentary Resv; DEx Veh, exercise vehicle; DEx Resv, exercise Resv. *P < .05.

**Figure 5.**
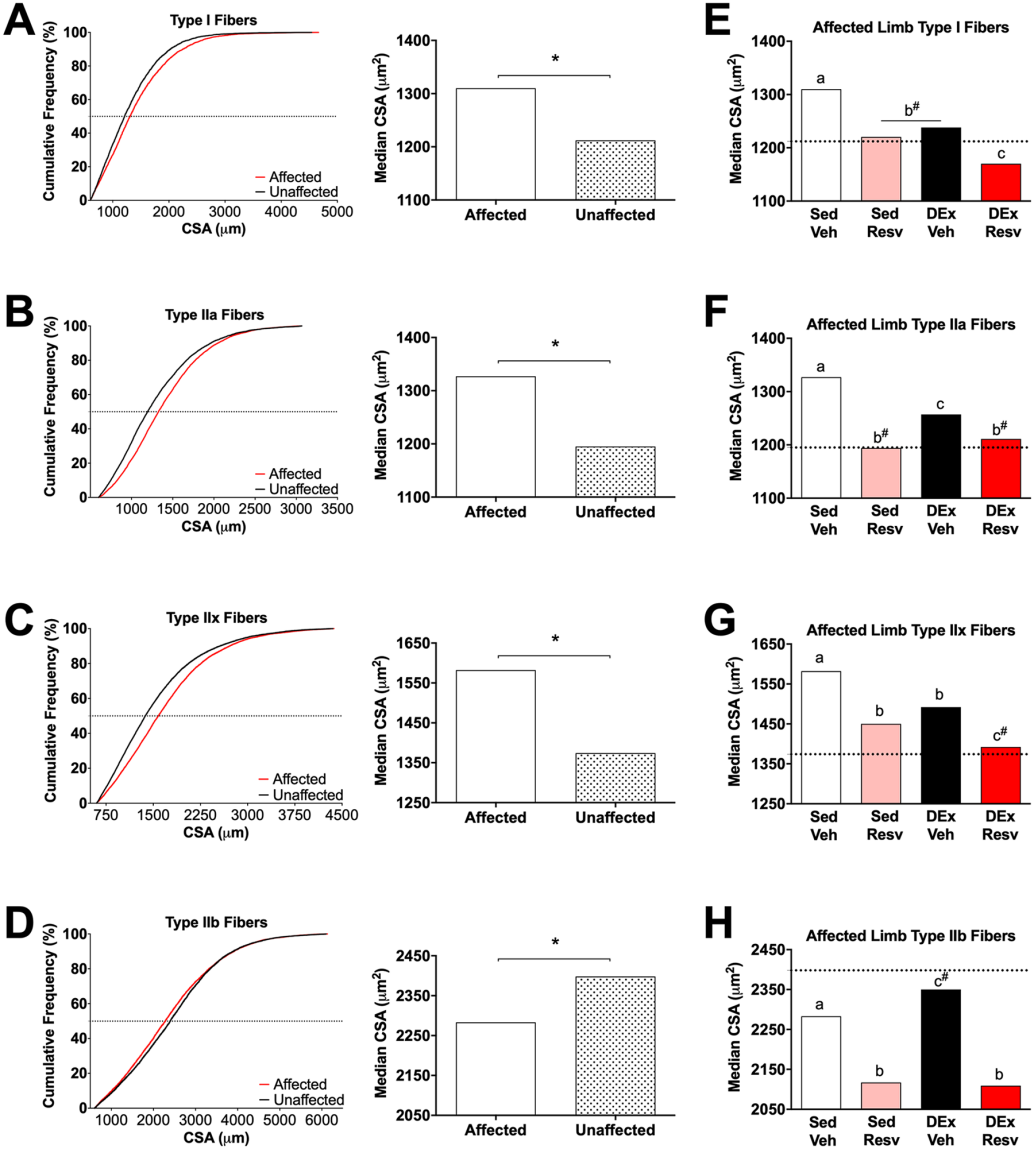
Stroke resulted in significant changes to muscle fiber cross-sectional area (CSA) that was improved with Resv and exercise. (A-D) Left panel: cumulative frequency distributions of the CSA of skeletal muscle fiber types revealed that the distribution of type I, IIa, IIx, and IIb fiber CSA was different between limbs in sedentary vehicle rats. The affected limb had larger type I, IIa, and IIx fibers and smaller type IIb fibers than the unaffected limb. Right panel: median CSA of each fiber type between limbs in sedentary vehicle. (E-H) Both Resv and exercise normalized changes to muscle fiber CSA. Data represent the median CSA of each muscle fiber type in the affected plantaris muscle. Dashed line, median fiber area in the unaffected plantaris muscle of sedentary vehicle (ie, control). Abbreviations: Resv, resveratrol; Sed Veh, sedentary vehicle control; Sed Resv, sedentary Resv; DEx Veh, exercise vehicle; DEx Resv, exercise Resv. ^a,b,c^ Represents groups with homogeneous frequency distributions; *P* > .05. ^#^ Indicates homogeneous frequency distribution with the unaffected plantaris muscle in sedentary vehicle (ie, control). *** *P* < .001. Fiber distributions were compiled from n = 8-9.

Muscle atrophy was apparent in the stroke-affected limb, whereby the CSA of the plantaris muscle from the affected limb was smaller than that of the unaffected limb (*F*_1,30_ = 5.238, *P* = .029; Figure 4C). The proportion of each muscle fiber type (I, IIa, IIx, IIb) was not different between limbs or in groups that received RESV or exercise rehabilitation (Figures 4E-4H; see Supplemental Results for statistics). In Sed Veh–treated rats the CSA of type I (*H*_1_ = 53.541, *P* < .001; Figure 5A), IIa (H_1_ = 221.690, *P* < .001; Figure 5B), and IIx (*H*_1_ = 442.664, *P* < .001; Figure 5C) muscle fibers were larger in the affected limb. In addition, the CSA of type IIb muscle fibers were smaller in the affected limb (*H*_1_ = 18.793, *P* < .001; Figure 5D).

### Both RESV and Exercise Mitigate Stroke-Induced Muscle Fiber Changes in the Affected Limb

Rats that received RESV following stroke had heavier plantaris muscles (*F*_1,62_ = 5.362, *P* = .024; Figure 4B). RESV also attenuated the difference in CSA of the plantaris muscle between the unaffected and affected limbs, albeit nonsignificantly (*F*_1,30_ = 3.696, *P* = .064; Figure 4D). The impact of each individual intervention on the distribution of muscle fiber CSA in the affected limb was compared with the unaffected limb of the vehicle-treated sedentary group (control limb). Type I fiber CSA was different between groups (*H*_4_ = 116.778, *P* < .0001; Figure 5E), with Sed RESV, DEx Veh, and the control limb having similar CSAs (*P* > .05). Sed Veh had the largest CSA (*P* < .05) and DEx RESV had the smallest CSA of type I fibers (*P* < .05). The CSAs of type IIa fibers were different between groups (*H*_4_ = 388.452, *P* < .0001; Figure 5F), with Sed RESV, DEx RESV, and the control limb being similar (*P* > .05) and Sed Veh and DEx Veh having larger CSAs (*P* < .05). Type IIx fibers were different between groups (*H*_4_ = 593.920, *P* < .0001; Figure 5G). DEx RESV had similar type IIx CSAs compared with the control limb (*P* > .05), whereas Sed RESV and DEx Veh, and Sed Veh had larger type IIx fibers (*P* < .05). Type IIb muscle fiber CSA was also different between groups (*H*_4_ = 329.815, *P* < .0001; Figure 5H), with DEx Veh having similar CSAs to the control limb (*P* > .05). Sed RESV and DEx RESV had the smallest CSAs of type IIb fibers, and the CSAs of IIb fibers in Sed Veh was different from all groups, displaying only a modest reduction in type IIb CSA (*P* < .05). In addition, RESV reduced vascular density in both the unaffected and affected limbs (see Supplemental Materials, Figure 3).

## Discussion

Stroke results in decreased cardiovascular fitness and skeletal muscle deconditioning, thereby limiting the capacity of individuals to engage in intensive rehabilitation.^8,11^ To date, these attributes have not been investigated in preclinical stroke models. Because mobility is an important concern for stroke survivors, we used a hindlimb model of stroke to determine whether such an injury would reduce cardiovascular fitness and alter skeletal muscle phenotype similar to what is observed in stroke survivors. We also investigated the novel strategy of combining RESV, an exercise mimetic drug, with delayed physical exercise to improve behavioral recovery and reduce cardiovascular and skeletal muscle deconditioning poststroke. Although no additive or synergistic effect of this combination was found, both interventions provided distinct benefits on recovery indicating the added value of this combinatorial approach.

Several previous studies have assessed short-term gait changes in rodent stroke models^29-32^; however, the present study is the first to measure gait changes longitudinally following stroke specifically targeted to the hindlimb motor cortex. Gait deficits observed in the affected limb included increased time in brake, stance, stride, and swing phases of gait as well as increased variability in stance width, suggesting that movement of the affected limb was slow, exaggerated, and inconsistent following stroke. Similar to gait deficits following middle cerebral artery (MCAO) stroke, paw area at peak stance, which is indicative of reduced ability to load the limb, was only transiently decreased. As in human stroke,^33^ we observed that compensatory gait strategies developed in the weeks following stroke, such as more time spent in shared stance (ie, dual support).

RESV attenuated early stroke-induced deficits because RESV-treated rats displayed fewer foot faults on the beam task 1 week following stroke. It is important to note that RESV given 24 hours poststroke had no effect on infarct volume, thereby ruling out any confounding neuroprotective effect. Similarly, RESV administration for 7 days beginning 24 hours after MCAO stroke reduces rotarod and tightrope walking deficits.^23^ Here, RESV also enhanced late stages of poststroke recovery, improving performance on the beam task from week 3 to week 6 poststroke and attenuating the development of compensatory gait strategies. Previous studies suggested that increased angiogenesis may be an important contributing factor to the recovery-enhancing effects of RESV^21,23,34^; however, in our study, an increase in angiogenesis was only apparent in the sedentary rats that received RESV. Because RESV acts in an endothelial nitric oxide-dependent manner, the behavioral benefit of RESV in other studies could also be a result of increased nitric oxide production or increased cerebral blood flow.^35^ However, quantifying nitric oxide production or a functional assessment of cerebral arterioles in peri-infarct tissue was beyond the scope of this study.

Although numerous preclinical studies report that aerobic exercise enhances neuroplasticity and recovery following stroke,^17^ very few of these studies use common human indices of aerobic capacity such as VO_2_ peak to characterize their exercise interventions. Instead, exercise frequently consists of unlimited access to a running wheel or some arbitrary time on a treadmill, which is difficult to relate to human exercise.^36^ Furthermore, no preclinical studies have incorporated resistance training as part of their exercise intervention, even though it is recommended for clinical use.^16,25^ Consequently, we sought to bridge this translational divide by utilizing both progressive aerobic exercise at a predefined intensity (70%-80% VO_2_ peak) and resistance exercise based on recommendations for stroke survivors.^25^ Our aerobic exercise consisted of forced treadmill use, which is very similar to human rehabilitation protocols. In addition, forced exercise has been shown to be more effective than voluntary exercise in animal studies.^17,37^ We used a delayed exercise intervention, beginning 10 days poststroke, to avoid possible injury exacerbating effects of very early exercise.^38^ In addition, the goal of this experiment was to determine whether administering RESV during the acute period poststroke, when patients are extremely inactive, would attenuate immediate reductions in cardiovascular fitness. Sedentary and exercise groups were not different at week 6 on the beam traversal task. However, after initiating the exercise rehabilitation, rats that exercised showed a significant reduction in foot faults from week 1 to week 3. The requisite timing and dose of exercise for enhancing recovery is not known; however, delaying exercise until 10 days following stroke is likely suboptimal. Indeed, some evidence suggests that in rodent models using forced exercise, the optimal time window for neuroprotection and recovery is between 1 and 5 days.^17,37^ Similarly, exercise did not increase cortical angiogenesis as reported elsewhere,^39,40^ but this too may require exercise to be initiated within 24 to 48 hours poststroke,^17^ which again may increase risk in humans.^38,41^

Compared to some studies,^22,42^ we did not observe a synergistic behavioral benefit of RESV and exercise. It is likely that the greater efficacy in other studies was because RESV was given in close temporal proximity to stroke during the period of reperfusion,^22,42^ thereby reducing infarct volume and introducing a confounding neuroprotective effect.^21,35,42,43^ Indeed, similar to our study, RESV has no effect on infarct volume if given 24 hours post-MCAO.^23^

Unlike previous work,^44-48^ stroke effects on skeletal muscle were assessed across the whole cross section rather than regions of interest. A novel open-source workflow to allow other researchers to fully and efficiently characterize changes to skeletal muscle fibers across an entire muscle cross section, or a muscle biopsy from humans, is provided. As in humans,^8,9,14^ muscular atrophy was evident in our rats’ stroke-affected limbs. Consistent with human stroke,^14^ hypertrophy of slow-twitch skeletal muscle fibers (I, IIa, and IIx) and atrophy of fast-twitch fibers (IIb) in the plantaris muscle from the affected limb were evident. In contrast to humans where muscle fibers shift to a greater number of fast-twitch type II muscle fibers in the muscle of the stroke-affected leg,^49,50^ we found no significant difference in the proportion of fiber type in the affected plantaris muscle. However, the reported change in fiber type proportions is usually observed in older, chronic stroke survivors whose comorbidities (eg, obesity and diabetes) can affect skeletal muscle phenotype.^51^ In summary, stroke targeting the rat hindlimb motor cortex resulted in muscular atrophy and changes in CSA of specific fiber types, similar to human patterns.

Although preclinical studies have identified a critical window poststroke when rehabilitation has the greatest efficacy,^2-5,52^ stroke patients are mostly sedentary during this period.^6,7^ RESV has shown promise in reducing fatigue and increasing measures of aerobic capacity.^19,20,53^ However, contrary to our hypothesis, RESV did not offset reductions in VO_2_ peak or improve exercise tolerance. It is possible that longer-term RESV administration is required, because in healthy animals, RESV needs to be administered for longer than 12 weeks to increase aerobic metabolism.^19,20^ Although a synergistic interaction between RESV and exercise likely exists, the relationship is not always additive, and our understanding of this interaction is incomplete.^54,55^ In the present study, RESV limited muscular atrophy within the plantaris muscle of affected limbs and increased plantaris muscle mass. In addition, hypertrophy of slow-twitch muscle fibers (I, IIa) following stroke was normalized in RESV rats suggesting that RESV may improve oxidative metabolism in stroke-affected muscles. Although we did not measure the oxidative capacity of each fiber type, an inverse relationship exists between skeletal muscle fiber area and oxidative capacity.^56^ Recent gain of function data in the mouse suggest that the amelioration of skeletal muscle atrophy is a result of SIRT1 signaling,^57^ which is activated by RESV.^20^ Although RESV did not improve aerobic capacity on a poststroke maximal exercise test, it led to changes in skeletal muscle congruent with behavioral benefits for stroke-affected muscle and, thus, warrants future investigation.

Prolonged inactivity is a significant barrier to poststroke rehabilitation and recovery because it rapidly decreases metabolic, muscular, and cardiovascular conditioning.^6,7,58^ Consequently, rehabilitation should include a combination of aerobic and resistance exercise to limit deconditioning and enhance neuroplasticity.^11,16,25^ Rats that engaged in exercise did not exhibit reductions in VO_2_ peak beyond what was initially observed poststroke. The fact that the stroke itself caused a reduction in cardiorespiratory fitness independent of disease comorbidities emphasizes the importance of rapidly implementing exercise therapy to reduce the risk of recurrent stroke.^59^ Exercise also normalized type I and IIb muscle fiber CSA and increased PGC1α protein content, which is indicative of increased aerobic capacity.

Moving forward, it is essential that preclinical research models capture key aspects of human stroke.^12,13^ Here, we describe a novel stroke model that includes clinically relevant gait deficits, decreases in cardiovascular fitness and exercise capacity, and skeletal muscle deconditioning. Notably, we found that RESV during the early acute phase, when stroke survivors are extremely inactive, reduced early behavioral deficits, decreased compensatory movements, improved long-term recovery, and mitigated changes to stroke-affected muscle. Our results clearly demonstrate the added value of combining exercise and drug interventions where, even in the absence of synergistic or additive effects, each component provided unique benefits important for recovery.

## Supplemental Material

sj-pdf-1-nnr-10.1177_15459683211005019 – Supplemental material for An Exercise Mimetic Approach to Reduce Poststroke Deconditioning and Enhance Stroke Recovery Click here for additional data file. Supplemental material, sj-pdf-1-nnr-10.1177_15459683211005019 for An Exercise Mimetic Approach to Reduce Poststroke Deconditioning and Enhance Stroke Recovery by Matthew W. McDonald, Matthew S. Jeffers, Lama Issa, Anthony Carter, Allyson Ripley, Lydia M. Kuhl, Cameron Morse, Cesar H. Comin, Bernard J. Jasmin, Baptiste Lacoste and Dale Corbett in Neurorehabilitation and Neural Repair

sj-txt-1-nnr-10.1177_15459683211005019 – Supplemental material for An Exercise Mimetic Approach to Reduce Poststroke Deconditioning and Enhance Stroke Recovery Click here for additional data file. Supplemental material, sj-txt-1-nnr-10.1177_15459683211005019 for An Exercise Mimetic Approach to Reduce Poststroke Deconditioning and Enhance Stroke Recovery by Matthew W. McDonald, Matthew S. Jeffers, Lama Issa, Anthony Carter, Allyson Ripley, Lydia M. Kuhl, Cameron Morse, Cesar H. Comin, Bernard J. Jasmin, Baptiste Lacoste and Dale Corbett in Neurorehabilitation and Neural Repair
