# Supplementary material for: An Exercise Mimetic Approach to Reduce Poststroke Deconditioning and Enhance Stroke Recovery
Source: Neurorehabil Neural Repair. 2021 Apr 7;35(6):471–85. doi: 10.1177/15459683211005019 (PMC8135250; doi:10.1177/15459683211005019)
Supplement: sj-pdf-1-nnr-10.1177_15459683211005019 – Supplemental material for An Exercise Mimetic Approach to Reduce Poststroke Deconditioning and Enhance Stroke Recovery [file sj-pdf-1-nnr-10.1177_15459683211005019.pdf]

Supplemental Materials for: An exercise mimetic approach to reduce post-stroke deconditioning and enhance stroke recovery. McDonald et al. 2021.

Methods

Dose-response pilot experiment. Strokes were induced via photothrombosis after a 12 hour fast. Briefly, rats were anesthetized (4% isoflurane induction, 2% isoflurane maintenance) injected with rose bengal through a lateral tail vein catheter (Sigma, R3877; 20mg/kg; dissolved in sterile water) and after 2 minutes, a cold light was illuminated for 10 minutes over the hindlimb sensorimotor cortex (AP -1.5 mm, ML +/- 3mm; relative to Bregma). Sham rats received only light illumination. Twenty-four hours after stroke rats received different doses of resveratrol (5mg/kg, 10mg/kg, 20mg/kg; i.p.) or vehicle (20% β-cyclodextrin, Toronto Research Chemicals Inc., H952565; 0.9% NaCl). The experiment lasted for 3 weeks, when skeletal muscle was extracted and investigated for PGC1α protein content in the soleus as described in the main experiment. Basal respiratory exchange ratios were determined using the OxyMax system (Columbus Instruments, USA).

Resistance exercise paradigm. For resistance exercise, rats climbed a vertical ladder (height: 1.1 m, incline: 80°, spacing between rungs: 2cm) with weights attached to the proximal portion of their tail.[2] Rats were familiarized pre-stroke to the weighted ladder climbing task over one week with incremental increases in weight (5 climbs with no additional weight and 5 climbs with 50% of their body mass). Between climbs, rats were allowed to rest for 2 minutes in a darkened box placed on top of the ladder. During a regular resistance training session, rats carried 50%, 75%, 90%, and 100% of their maximal lifting capacity for the first four climbs. Subsequent climbs were performed at 100% of their maximal lifting capacity until rats reached exhaustion (unable to finish a climb despite tactile stimulation of haunches). To determine maximal lifting capacity, rats were required to climb with a load of 75% of their body weight on the initial climb. Approximately thirty grams of weight was then added to each subsequent

climb until rats reached exhaustion. The weight carried prior to exhaustion was recorded as the new maximal lifting capacity. Maximal lifting capacity was determined every 4 exercise sessions.

Maximal exercise test. Rats were habituated to the enclosed treadmill over 5 days by incrementally increasing the workload and duration of time spent in the chamber. Initially, a steel grid at the back of the treadmill delivered an electric stimulus (0.2mA, 1 stimulus of 200ms every second) to encourage running. At the conclusion of each session, rats were rewarded with dark chocolate (100mg). Over time, the electrical stimulus was no longer required and was turned off to minimize stress. To calculate VO_2 peak, rats acclimated to the treadmill chamber for 5 minutes, followed by a warmup period of 5 minutes at 15m/min and 0% incline. Subsequently, the incline was increased to 15% and treadmill speed was increased 2m/min every 2 minutes until exhaustion. The criteria for reaching VO_2 peak was a leveling-off of oxygen consumption despite an increase in workload, a respiratory exchange ratio of 1.04 or higher, or when the rat could no longer maintain a sufficient pace (remaining at the back for longer than 3 seconds). Ambient air was delivered to the chamber at a rate of 4.5ml/min and chamber air (200ml/min) was sampled every 30 seconds by oxygen and carbon dioxide sensors.

Vascular density quantification in peri-lesional cortex. Z-projections were created from approximately thirty optical sections imaged at 20x magnification and spaced 0.5 microns apart. A Gaussian smoothing filter with a standard deviation of 0.5 microns was applied to z-projection images. Blood vessels were then identified using a local adaptive thresholding operation. Pixels were classified as a blood vessel if their intensity was larger than the average intensity inside a circular region centered at the pixel. The radius used for the circular region was 20 microns. The resulting image contained only two colors: white, representing blood vessels, and black, representing the background. White connected components smaller than 100 microns^2 were removed from the image. The medial lines of the blood vessels were then identified using the Palágyi-Kuba thinning procedure. Pixels in a medial line having only one neighbor

were associated with termination points and pixels with three or more neighbors were associated with bifurcation points.[1] The blood vessels were partitioned into a set of segments, where a segment was defined as a blood vessel between two termination or bifurcation points. Segments smaller than 3 microns were iteratively removed from the image.

Muscle fibres and CD31 labeled capillaries quantification. To identify each muscle fibre type and CD31 labeled capillaries, the neural network software Ilastik (www.ilastik.org) was used to produce pixel segmentation maps that were a binary representation of each fluorescent channel (Figure 1, supplemental materials). Prior to batch processing of images, Ilastik was manually trained to differentiate each muscle fibre type (I, IIa, IIb) and capillaries (CD31) in 10-15 images of plantaris muscle cross sections. To validate the accuracy of Ilastik to detect each fibre type, a separate 50 cross sectional images of plantaris muscle were manually counted and compared to Ilastik segmentations (Figure 2C, supplemental materials). Validation was also completed to establish the accuracy of Ilastik to delineate the cross-sectional area of each fibre type (Figure 2D, supplemental materials). In 68 plantaris muscles a 700µm x 700µm grid was placed over fluorescence images and within every 32nd counting grid each fibre type was manually traced. Manual tracing of the cross-sectional area of each fibre type within the grid was compared to the accuracy of Ilastik to segment out the same muscle fibres. To quantify CD31 labeled capillaries, a similar process was conducted (Figure 2E, supplemental materials). A 700µm x 700µm counting grid was placed on fluorescence images from 70 plantaris muscle cross-sections and within every 32nd counting grid each capillary was manually counted and compared to Ilastik segmentations. Quantification of pixel segmentation maps to determine skeletal muscle fibre counts, cross-sectional area (CSA), and vascular density was conducted using a custom ImageJ (NIH, USA) script (found in supplemental materials; MuscleFiberTyping.txt).

Western blotting. The red portion of the tibialis anterior muscle from the affected limb was homogenized in a 1:5 (w/v) ratio of lysis buffer (10mM Tris-HCl, 150mM NaCl, 1% Triton-X 100, 1% glycerol) with protease inhibitor cocktail (Roche), centrifuged for 15 minutes at 4ºC, and protein concentrations in supernatant were determined with a Bradford protein assay. Supernatant was then mixed with buffer (1M Tris-HCl, 40% glycerol, 0.04% SDS, 0.04% bromophenol blue, 5% β-mercaptoethanol) and loaded into TGX Stain-Free Protein Gels (BioRad; Mississauga, Ontario). Gel electrophoresis was performed in running buffer (25mM Tris-HCl, 200mM glycine, 0.1% SDS) at 40V until proteins were through the stacking gel, followed by 80V until proteins were fully separated. Proteins were then transferred to nitrocellulose membranes at 110V at 4ºC in transfer buffer (25mM Tris-HCl, 192mM glycine, 20% methanol (m/v)). Membranes were blocked in 5% nonfat dry milk in Tris-buffered saline (TTBS; 10mM Tris-HCl, 100mM NaCl, 0.1% Tween-20) for 1 hour, followed by overnight incubation in PGC-1α (NBP1-04676; Novus Biologicals, USA) primary antibody at 1:2000 in 2% TTBS. The following day membranes were washed in TTBS and incubated with secondary antibody (#1706515; BioRad; Mississauga, Ontario) at 1:2000 for 1 hour. Membranes were developed using chemiluminescent detection (BioRad, Mississauga, Ontario; Western C Enhanced Chemiluminescent Kit, 170-5070) and imaged using a BioRad Chemidoc XRS imager and optical densities were quantified using BioRad Quantity One software. Equal loading was normalized to total proteins on the membrane as detected using the Stain-free technology (BioRad; Mississauga, Ontario).

Results

Dose-response pilot experiment. There was a trend that 5mg/kg of Resv reduced the basal RER of rats in stroked rats ($p=0.055$; see supplemental Figure 1). PGC1α protein content was increased in rats that had a stroke and received 5mg/kg of Resv compared to sham ($p<0.05$; see supplemental Figure 1C).

Similarly, there was a trend that PGC1α protein content was increased in rats that had a stroke and received 5mg/kg of Resv compared to vehicle-treated rats (p=0.053).

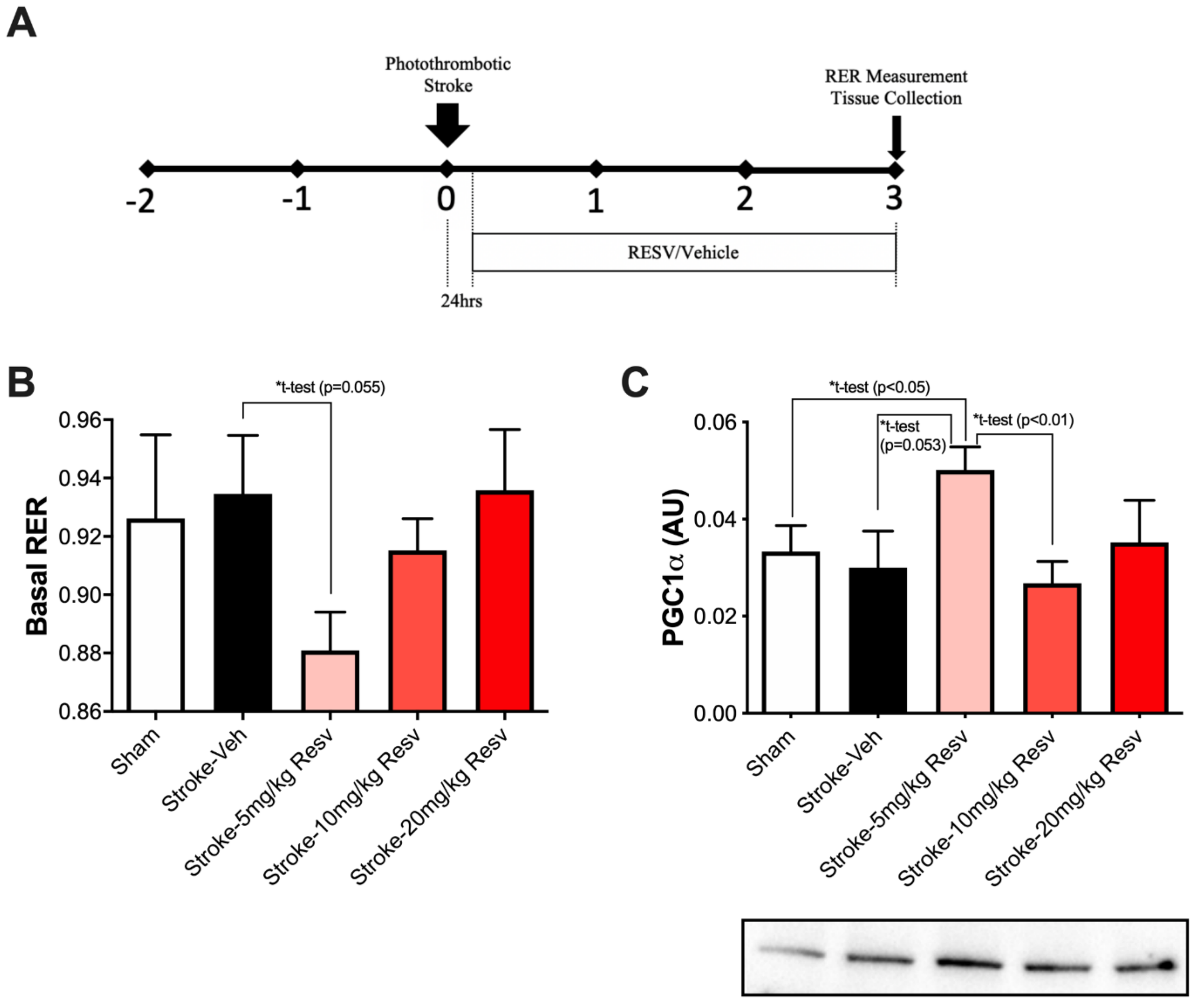

Supplemental Figure 1. Dose-response pilot experiment (n=5). (A) Experimental timeline. (B) Respiratory exchange ratio (RER) at rest at the completion of the experiment. (C) Peroxisome proliferator-activated receptor gamma coactivator 1-α (PGC1α) in the plantaris muscle. Data are a mean ± standard error.

Validation of skeletal muscle fibre quantification workflow. Manual counts of type I ($r=0.83$, $r^2=0.69$, $p<0.0001$; Supplemental Figure 2C, *left panel*), IIa ($r=0.90$, $r^2=0.81$, $p<0.0001$; Supplemental Figure 2C, *middle panel*), and IIb ($r=0.86$, $r^2=0.75$, $p<0.0001$; Supplemental Figure 2C, *right panel*) were significantly correlated with automated counts. Manual tracing of type I ($r=0.83$, $r^2=0.68$, $p<0.0001$; Supplemental Figure 2D, *left panel*), IIa ($r=0.90$, $r^2=0.82$, $p<0.0001$; Supplemental Figure 1D, *middle panel*), and IIb ($r=0.81$, $r^2=0.66$, $p<0.0001$; Supplemental Figure 2D, *right panel*) were significantly correlated with automated area detection. Manual counting of capillaries within regions of interest was significantly correlated ($r=0.89$, $r^2=0.80$, $p<0.0001$; Supplemental Figure 2E, *middle panel*) with automated capillary detection.

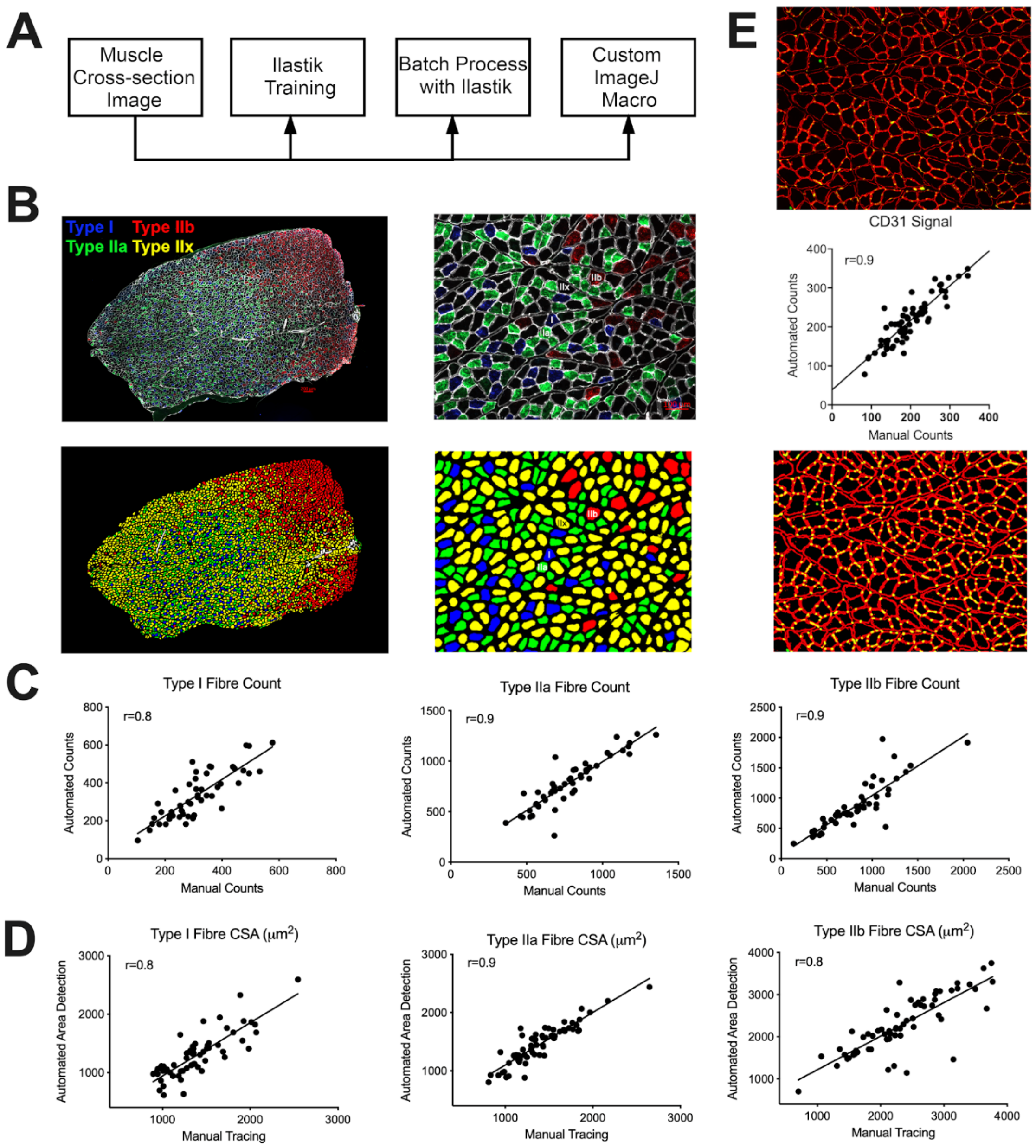

Supplemental Figure 2. Validation of workflow for automated quantification of skeletal muscle fibres. (A) For each colour channel of the fluorescent image (i.e. each fibre type, capillary), Ilastik was trained to classify each pixel as signal. Subsequently, fluorescent images were batch processed using Ilastik and binary pixel classification maps were processed through a custom ImageJ/Fiji script. Fibres not quantified as being either type I, IIa, or IIb, were considered IIx fibres. (B) *Top panel;* Representative image of plantaris muscle. *Lower panel*; Representative image of muscle fibre types quantified using workflow. (C) Correlation between manual vs. automated counts of each fibre type. (D) Correlation between manual vs. automated delineation of each fibre area. (E) *Upper panel;* Representative image of CD31 labeled capillaries (yellow) and muscle fibres identified with laminin (red). *Middle panel;* Correlation between manual vs. automated counts of capillaries. *Lower panel;* Representative image of muscle capillaries types quantified using workflow.

Digigait results. The duration of time the limb spent in stance ($F_{2,48}$ = 7.489, p=0.001), increased at week 1 post-stroke (t_{27} = 3.265, p=0.009) and remained elevated at week 6 (t_{27} = 2.668, p=0.038). The time the affected limb spent in the swing phase ($F_{1.53,36.73}$ = 9.627, p=0.001), increased at week 1 post-stroke (t_{27} = 5.291, p<0.001) and week 6 (t_{27} = 3.180, p=0.011) compared to pre-stroke. The length of time that the affected limb was in the brake stage of gait ($F_{2, 48}$ = 9.166, p<0.001), increased at week 1 (t_{27} = 4.978, p<0.001) and week 6 (t_{27} = 3.997, p=0.001) following stroke. The stride phase of gait ($F_{2, 48}$ = 19.422, p<0.001) was increased 1 week (t_{27} = 4.697, p<0.001) and at 6 weeks (t_{27} = 6.364, p<0.001) following stroke. Stride length of the affected limb during gait ($F_{2, 48}$ = 19.493, p<0.001) was increased at week 1 (t_{27} = 4.756, p<0.001) and at 6 weeks (t_{27} = 6.461, p<0.001) post-stroke. Similarly, the speed at which rats loaded the affected limb during gait ($F_{2, 48}$ = 16.099, p<0.001) was reduced at week 1 (t_{27} = 5.032, p<0.001) and week 6 (t_{27} = 4.232, p<0.001). In regards to the time the affected limb spent in shared stance (i.e. dual support), there was an interaction between time post-stroke and Resv treatment ($F_{2, 35.32}$ = 3.570, p=0.050).

Proportion of each muscle fibre type (I, IIa, IIx, IIb). The proportion of type I muscle fibres were not different between limbs (F1, 30 = 0.495, p=0.487, Figure 4E), or between rats given Resv (F1, 30 = 2.015, p=0.166) or exercise (F1, 30 = 0.054, p=0.818). Type IIa muscle fibre proportions were similar between limbs (F1, 30 = 1.724, p=0.199, Figure 4F), independent of Resv (F1, 30 = 0.044, p=0.836) or exercise (F1, 30 = 0.517, p=0.478). The percentage of type IIx were similar between experimental groups independent of limb (F1, 30 = 0.098, p=0.756, Figure 4G), Resv (F1, 30 = 0.205, p=0.654), or exercise (F1, 30 = 0.427, p=0.518). The proportion of type IIb muscle fibres were also similar between limbs (F1, 30 = 0.431, p=0.516, Figure 4H), and between rats treated with Resv (F1, 30 = 0.851, p=0.364) or exercise (F1, 30 = 0.048, p=0.827).

Vascular density in the plantaris muscle. Resv also had an effect on skeletal muscle vascular density, where vasculature density was reduced in the plantaris muscle independent of limb ($F_{1,\ 31}$ = 9.015, p=0.005, Supplemental Figure 3).

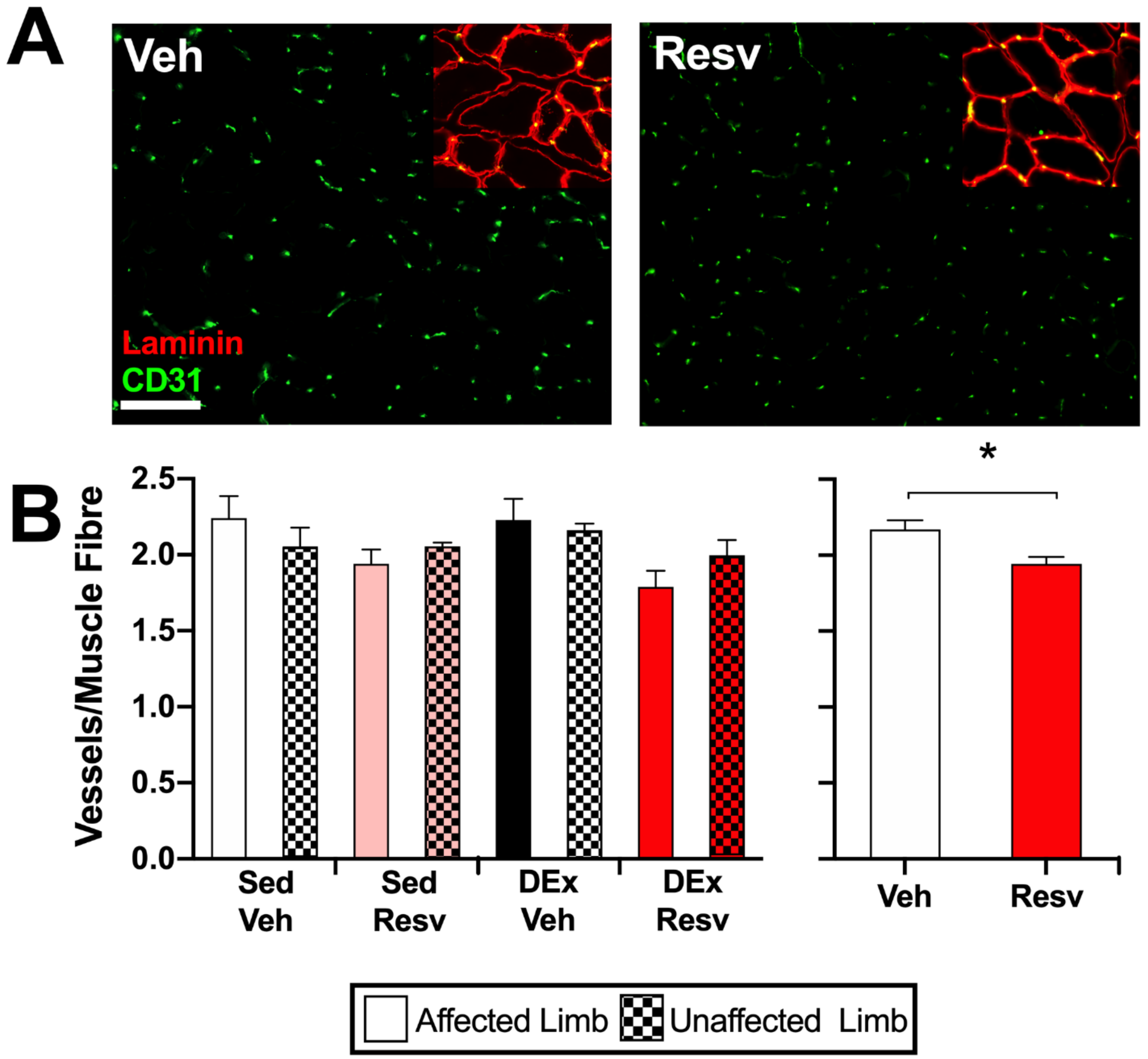

Supplemental Figure 3. Resveratrol reduced vascular density in the plantaris muscle. (A) *Left panel;* Representative images of CD31 (green) in the plantaris in vehicle treated rats. *Right panel;* Representative images of CD31 (green) in the plantaris in Resv treated rats. *Inset,* Laminin (red) to differentiate muscle fibers. 100µm scale bar. (B) *Left panel,* Vessels per muscle fiber (n=8-9). *Right panel;* Resveratrol decreased the density of vasculature independent of limb or exercise rehabilitation (n= 34-36). *p<0.05. Data are a mean ± standard error.

References.
1. Palàgyi K, Kuba A. A 3D 6-subiteration thinning algorithm for extracting medial lines. *Pattern Recognit. Lett.* 1998;19:613–627.
2. McDonald MW, Olver TD, Dotzert MS, et al. Aerobic exercise training improves insulin-induced vasorelaxation in a vessel-specific manner in rats with insulin-treated experimental diabetes. *Diabetes Vasc. Dis. Res.* 2019;16:77–86.
